# Suppression of triple-negative breast cancer aggressiveness by LGALS3BP via inhibition of the TNF-α–TAK1–MMP9 axis

**DOI:** 10.1038/s41420-023-01419-9

**Published:** 2023-04-11

**Authors:** Eun-Gene Sun, Veena Vijayan, Mi-Ra Park, Kyung Hyun Yoo, Sang-Hee Cho, Woo-Kyun Bae, Hyun-Jeong Shim, Jun-Eul Hwang, In-Kyu Park, Ik-Joo Chung

**Affiliations:** 1 Department of Hematology and Oncology, Chonnam National University Medical School and Hwasun Hospital, Hwasun, Republic of Korea; 2 Immunotherapy Innovation Center, Chonnam National University Medical School and Hwasun Hospital, Hwasun, Republic of Korea; 3 Department of Biomedical Sciences and Center for Global Future Biomedical Scientists at Chonnam National University, Chonnam National University Medical School, Gwangju, Republic of Korea; 4 Department of Biological Science, Sookmyung Women’s University, Seoul, Republic of Korea; 5 Combinatorial Tumor Immunotherapy MRC Center, Chonnam National University Medical School, Hwasun, Republic of Korea

**Keywords:** Breast cancer, Breast cancer

## Abstract

Transforming growth factor-β-activated kinase 1 (TAK1), which is highly expressed and aberrantly activated in triple-negative breast cancer (TNBC), plays a pivotal role in metastasis and progression. This makes it a potential therapeutic target for TNBC. Previously, we reported lectin galactoside-binding soluble 3 binding protein (LGALS3BP) as a negative regulator of TAK1 signaling in the inflammatory response and inflammation-associated cancer progression. However, the role of LGALS3BP and its molecular interaction with TAK1 in TNBC remain unclear. This study aimed to investigate the function and underlying mechanism of action of LGALS3BP in TNBC progression and determine the therapeutic potential of nanoparticle-mediated delivery of LGALS3BP in TNBC. We found that LGALS3BP overexpression suppressed the overall aggressive phenotype of TNBC cells in vitro and in vivo. LGALS3BP inhibited TNF-α-mediated gene expression of matrix metalloproteinase 9 (*MMP9*), which encodes a protein crucial for lung metastasis in TNBC patients. Mechanistically, LGALS3BP suppressed TNF-α-mediated activation of TAK1, a key kinase linking TNF-α stimulation and *MMP9* expression in TNBC. Nanoparticle-mediated delivery enabled tumor-specific targeting and inhibited TAK1 phosphorylation and *MMP9* expression in tumor tissues, suppressing primary tumor growth and lung metastasis in vivo. Our findings reveal a novel role of LGALS3BP in TNBC progression and demonstrate the therapeutic potential of nanoparticle-mediated delivery of LGALS3BP in TNBC.

## Introduction

Breast cancer (BC) is the leading cause of cancer-related deaths in women worldwide [1, 2]. BC is highly heterogeneous and categorized based on the expression of the estrogen receptor, progesterone receptor, and human epidermal growth factor receptor [2]. There is no targeted therapy for TNBC owing to the lack of expression of these receptors. Although chemotherapy remains the standard treatment, 60% of TNBC patients develop tumors resistant to chemotherapy [3, 4]. Thus, there is an urgent need to identify novel targets and therapeutic strategies for TNBC.

Transforming growth factor-β-activated kinase 1 (TAK1) is a key player in responses against various cytokines and stimulators, including Wnt [5], BMP [6], TNF-α, IL-1β [7], and TGF-β [8], which are critical mediators of the inflammatory response and tumor progression, thereby providing a molecular link between inflammation, fibrosis, and carcinogenesis. Increasing evidence has revealed the critical role of TAK1 in TNBC progression. TAK1 overexpression and aberrant activation are characteristic features of TNBC. TAK1 expression is upregulated in up to 30% of BC cases and more highly expressed and robustly activated in TNBC than in the other subtypes [9–11]. TGF-β induces the production of the alternative splicing isoform of TAK1 in TNBC; the TAK1 isoform is constitutively active and supports TGF-β-induced epithelial-to-mesenchymal transition and nuclear factor-kappa B (NF-κB) signaling [12]. Furthermore, TAK1 promotes TNBC lung metastasis [10]. Overall, these findings indicate TAK1 as a potential therapeutic target for TNBC.

The lectin galactoside-binding soluble 3 binding protein (LGALS3BP) is a multifunctional glycoprotein involved in immune responses, defense against viral infections, and cancer progression [13]. Its role in cancer progression has been widely studied. However, evidence regarding its role in cancer progression remains controversial. We have previously shown that LGALS3BP inhibits the inflammatory response by negatively regulating TAK1–NFκB signaling in macrophages and mouse embryonic fibroblasts by inhibiting TAK1 phosphorylation and kinase activity via protein–protein interactions [14]. LGALS3BP controls colon homeostasis by suppressing inflammation [15]. LGALS3BP exhibits antitumor activity in colorectal cancer via β-catenin ubiquitination, and high LGALS3BP expression is associated with better overall survival in colon cancer patients [16, 17]. However, the specific role of LGALS3BP in TNBC remains to be elucidated.

Nano-delivery systems have been extensively utilized for drug delivery in cancer treatment. Biological barriers, including enzymatic degradation in the blood and limited membrane crossing, hinder drug delivery. To overcome these limitations, liposomes have been used as delivery systems in pre-clinical and clinical applications [18–20]. Liposomes provide safe, repeatable, and enhanced drug delivery with minimal side effects. Passive targeting of liposomes can be converted into active targeting by conjugating peptides, ligands, and antibodies. The cyclic arginine-glycine-aspartic acid (RGD) peptide is a specific ligand for integrin receptors that are overexpressed in most tumor types [21]. Therefore, cRGD-conjugated liposomes are an effective nano-delivery system to improve tumor targeting for cancer treatment [22–24].

In this study, we aimed to examine the role of LGALS3BP and the mechanisms underlying its action in the aggressiveness and progression of TNBC. Furthermore, we attempted to deliver LGALS3BP into TNBC cells and verify its therapeutic effects on TNBC progression and lung metastasis.

## Results

### LGALS3BP inhibits TNBC cell proliferation, migration, and invasion

**Fig. 1:**
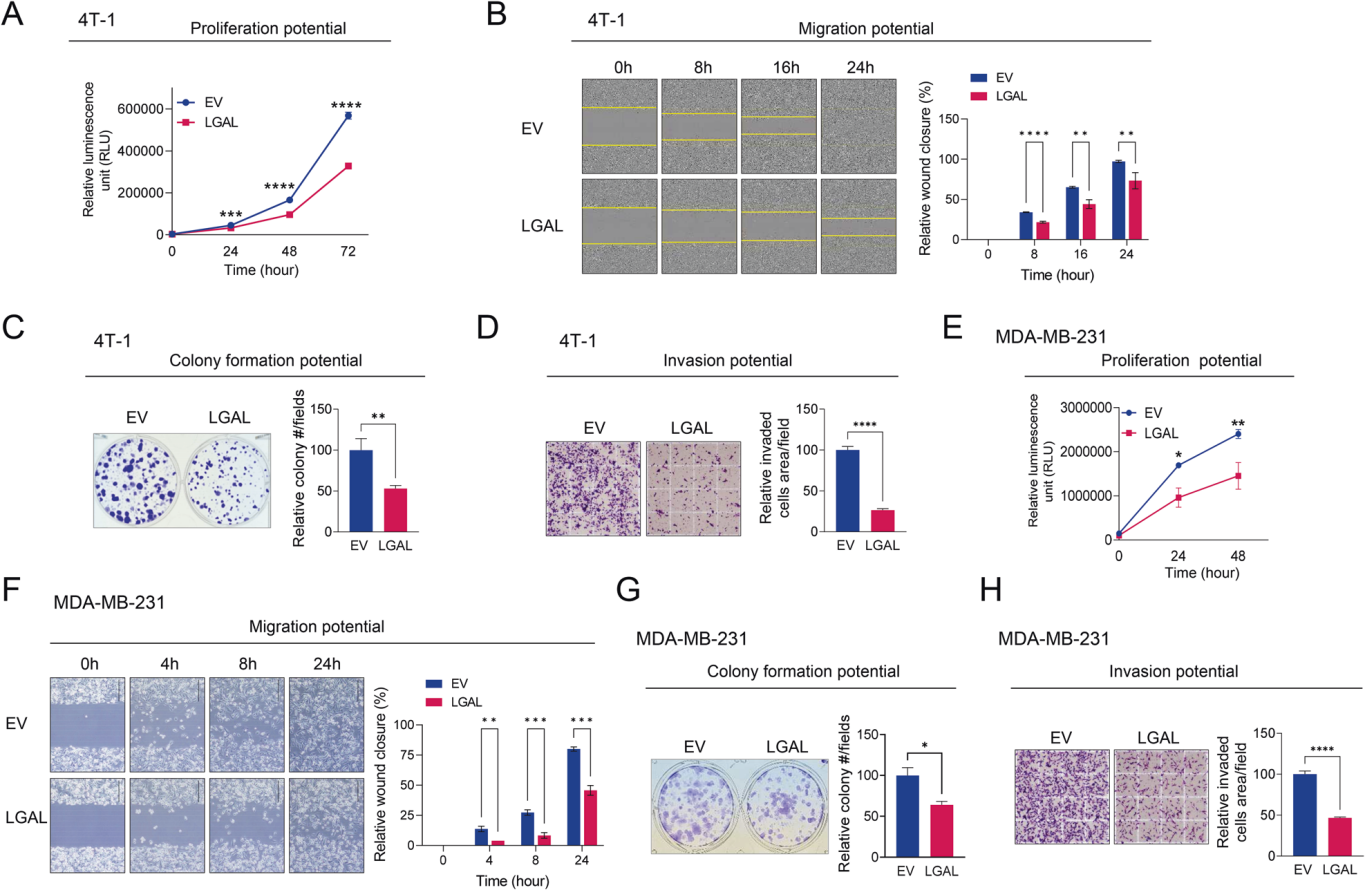
LGALS3BP overexpression inhibits the overall aggressiveness of TNBC cells. **A** Comparison of the proliferation potential of 4T-1 cells stably overexpressing EV or LGALS3BP. Proliferation ability was determined via the Realtime-Glo MT cell viability assay. **B**–**D** Migration, survival, and invasion potential of 4T-1 cells overexpressing EV or LGALS3BP were compared using a wound-healing assay (**B**), colony-forming assay (**C**), and Transwell invasion assay (**D**). **E** Comparison of the proliferation potential of MDA-MB-231 cells overexpressing EV or LGALS3BP. **F**–**H** Comparison of the migration (**F**), survival (**G**), and invasion (**H**) potential of MDA-MB-231 overexpressing EV or LGALS3BP, determined using wound-healing, colony-forming, and Transwell invasion assays, respectively. Data are presented as mean ± SD. *p < 0.05; **p < 0.01; ***p < 0.001; ****p < 0.0001. EV empty vector, LGAL LGALS3BP.

To verify the role of LGALS3BP in TNBC aggressiveness, we evaluated the oncogenic properties of EV- and LGALS3BP-OE TNBC cells. LGALS3BP overexpression markedly inhibited cell proliferation (Figs. 1A, E, S1) and significantly reduced the colony-formation potential (Fig. 1C, G), migration (Fig. 1B, F), and invasion abilities (Fig. 1D, H) of 4T-1 and MDA-MB-231 cells. These findings confirm that LGALS3BP inhibits various oncogenic properties of TNBC cells, including their growth, survival, migration, and invasiveness.

### LGALS3BP suppresses tumor growth and metastasis of TNBC in vivo

**Fig. 2:**
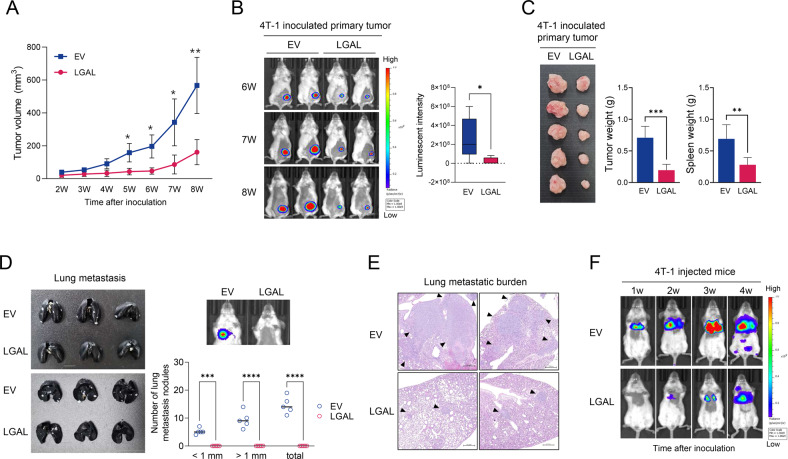
LGALS3BP suppresses in vivo tumor growth and lung metastasis of 4T-1 cells. Luciferase-labeled 4T-1 cells overexpressing EV or LGALS3BP were inoculated into the mammary fat pad of BALB/c mice (*n* = 5 /group) (**A**–**E**). **A** Comparison of the tumor-growing potential of luciferase-labeled 4T-1 cells overexpressing EV or LGALS3BP. **B** Representative in vivo bioluminescence images of tumor growth and quantification of bioluminescence signal. **C** Comparison of the primary tumor weight and spleen weight. **D**, **E** Comparison of lung metastasis. Representative gross photographs of India ink-stained lungs and quantification of lung metastasis modules (**D**) and H&E-stained lung sections (**E**) obtained from BALB/c mice injected with luciferase-labeled 4T-1 cells overexpressing EV or LGALS3BP after 8 weeks. **F** Representative in vivo bioluminescence images of lung metastasis after intravenous injection of luciferase-labeled 4T-1 cells overexpressing EV or LGALS3BP. Data are presented as mean ± SD. *p < 0.05; **p < 0.01; ***p < 0.001; ****p < 0.0001.

LGALS3BP inhibited in vivo tumor growth in the orthotopic 4T-1 mouse model (Fig. 2A, B). Mice overexpressing LGALS3BP showed decreased primary tumor weight, spleen weight, and TNBC metastasis from the primary tumor to the lung (Fig. 2C, D). The tumor burden of 4T-1/LGALS3BP-OE-inoculated mice was lower than that of the 4T-1/EV-inoculated mice (Fig. 2E). In a spontaneous-metastasis mouse model, LGALS3BP overexpression reduced the lung metastatic potential of 4T-1 cells injected into mice (Fig. 2F). In vivo analysis confirmed the suppressive function of LGALS3BP in TNBC tumor progression (Fig. S2). These findings suggest that LGALS3BP overexpression suppresses primary tumor growth and lung metastasis of TNBC in vivo.

### LGALS3BP negatively regulates oncogenesis-related genes in TNBC cell lines

**Fig. 3:**
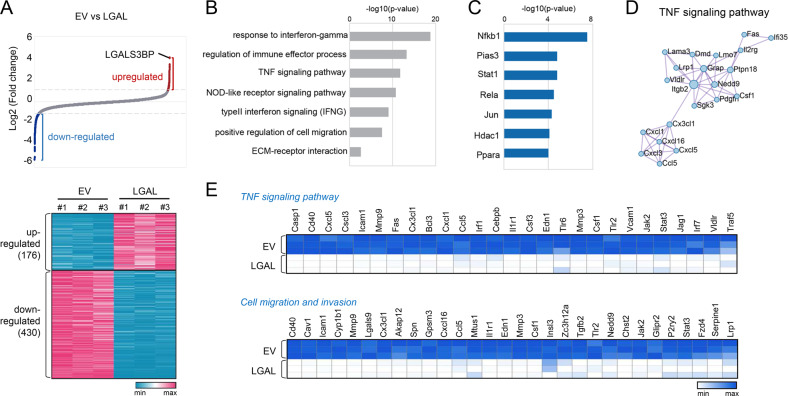
Transcriptome analysis of Lgals3bp-overexpressed cells. **A** Waterfall plot and heatmap showing the DEGs in Lgals3bp-overexpressed 4T-1 cells compared to controls (*n* = 3 /group). DEGs were defined as genes with over 2-fold expression in Lgals3bp-overexpressed cells compared to controls. **B** GO enrichment analysis of the biological process terms associated with 430 downregulated genes. **C** Representative results of the enrichment analysis by TRRUST (transcriptional regulatory relationships revealed via sentence-based test-mining). **D** PPI results of genes related to the TNF signaling pathway. **E** Heatmap depicting the expression of genes related to the TNF signaling pathway and cell migration and invasion.

To examine how LGALS3BP regulates TNBC aggressiveness, we performed RNA-sequencing in 4T-1 cells with overexpressed LGALS3BP. We identified 176 upregulated and 430 downregulated genes in LGALS3BP-overexpressed cells compared to those in EV controls (Fig. 3A). Downregulated genes via LGALS3BP were associated with response to interferon-γ, regulation of immune effector process, TNF signaling pathway, positive regulation of cell migration, and ECM-receptor interaction (Fig. 3B). Transcriptional regulatory relationships revealed via sentence-based test-mining (TRRUST) enrichment analysis showed that the downregulated genes were regulated by Nfkb1, Pias3, Stat1, Rela, Jun, Hdac1, and Ppara (Fig. 3C). Genes associated with the TNF signaling pathway strongly interacted with each other (Fig. 3D). The expression of genes such as *Mmp9*, *Mmp3*, and *Icam1*, which are involved in the TNF signaling pathway and cell migration, was remarkably reduced in LGALS3BP-overexpressing cells (Fig. 3E). LGALS3BP overexpression downregulated the mRNA expression of *TNF-α*, *Nfkb*, and mmp9, key players in TNBC aggressiveness and metastasis.

### LGALS3BP inhibits TNFα-mediated invasion of TNBC cells by suppressing the TAK1–NFκB–MMP9 axis

**Fig. 4:**
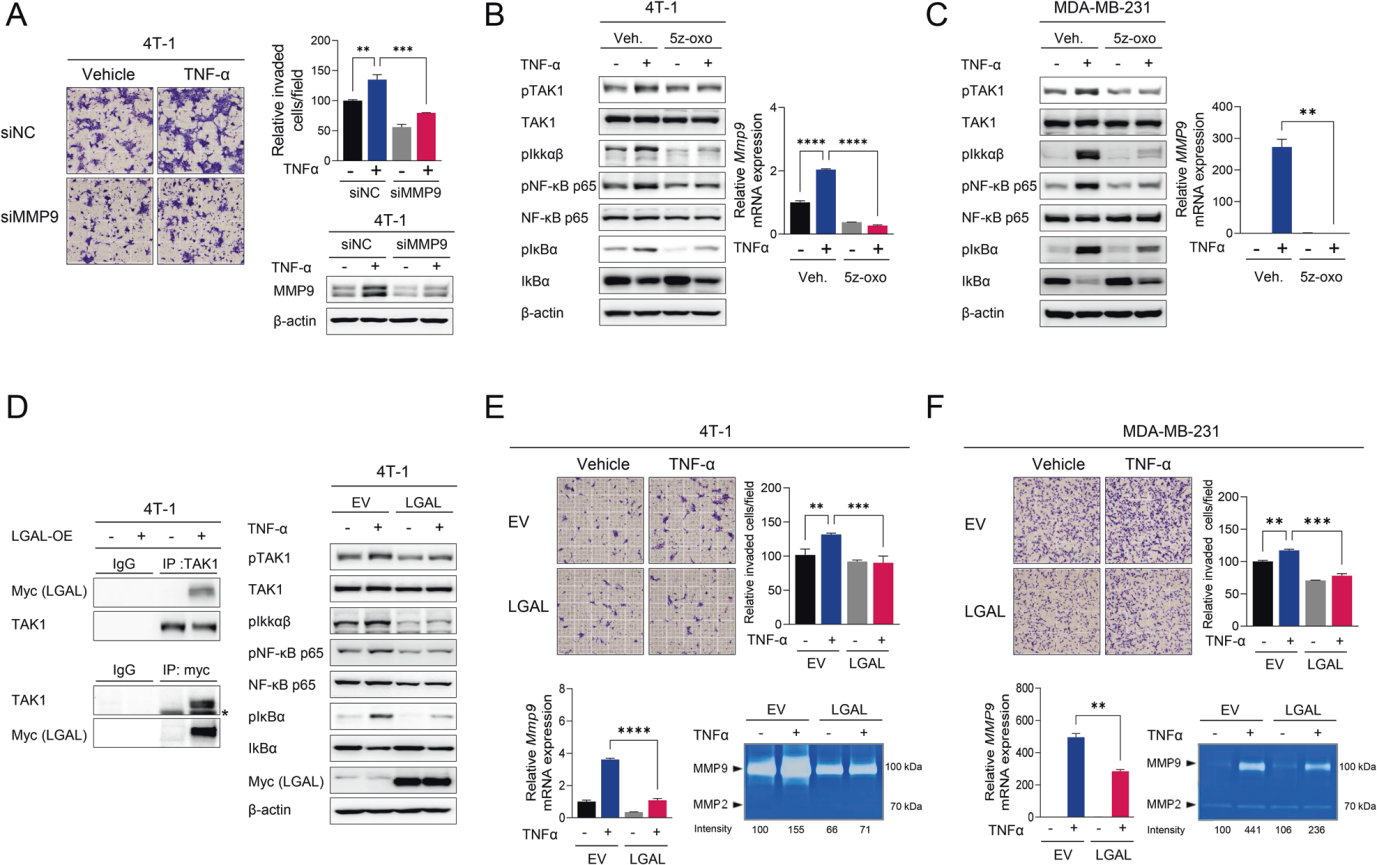
LGALS3BP inhibits TNFα-mediated invasion by suppressing the TAK1–NFκB–MMP9 axis. **A** Comparison of the invasion potential of 4T-1 cells with and without TNF-α or si-MMP9 treatment by the Transwell invasion assay. MMP9 expression was determined using western blotting analysis. **B**, **C** Effect of 5Z-7-oxozeaenol, TAK1 inhibitor, on TNF-α-mediated activation of TAK1–NFκB signaling and *MMP9* expression in TNBC cells. **B** 4T-1, (**C)** MDA-MB-231. The TNF-α-related signaling pathway was determined using western blotting analysis using the indicated Abs. *MMP9* mRNA expression was determined using RT-qPCR. **D** Reciprocal co-IP between Myc-tagged LGALS3BP, TAK1, and western blot. Left: Co-IP with anti-Myc or TAK1 antibodies; subsequent immunoblotting analyses validate the binding of LGALS3BP to endogenous TAK1 in TNBC cells. Asterisks (*) indicate a non-specific band. Right: Western blotting analysis showing TNF-α-related TAK1–NFκB signaling. **E**, **F** Effect of LGALS3BP overexpression on TNF-α-mediated invasiveness, *MMP9* expression, and MMP9 secretion in TNBC cells. **E** 4T-1, **F** MDA-MB-231. Upper: Transwell invasion assay and quantification. Lower: RT-qPCR and zymography for determination of MMP9 gene expression and secretion, respectively. Data are presented as mean ± SD. *p < 0.05; **p < 0.01; ***p < 0.001; ****p < 0.0001.

TNF-α treatment significantly increased the invasive potential of TNBC cells with increasing *Mmp9* expression; *Mmp9* knockdown suppressed TNF-α-induced invasion (Fig. 4A). TNF-α treatment induced TAK1–IKKαβ–NF-κB signaling cascade phosphorylation and the expression of *Mmp9*, a target of NF-κB. These effects were significantly suppressed by treatment with 5Z-7-oxozeaenol (5Z-oxo), an irreversible inhibitor of TAK1, in 4T-1 and MDA-MB-231 TNBC cells (Fig. 4B, C). These findings indicate that TNF-α promotes invasiveness by activating TAK1 signaling and increasing *MMP9* expression. TAK1 acts as a link between TNF-α stimulation and *MMP9* expression.

LGALS3BP interacted directly with TAK1, and LGALS3BP overexpression inhibited TNF-α-mediated phosphorylation of TAK1, IKKαβ, NF-κB, and IκB (Figs. 4D, S4). Based on its role in TAK1 signaling, we investigated whether LGALS3BP suppresses TNF-α-mediated invasiveness and *MMP9* expression in TNBC cells. LGALS3BP overexpression significantly suppressed TNF-α-mediated invasiveness in 4T-1 and MDA-MB-231 cells and inhibited TNF-α-mediated MMP9 expression and production in TNBC cells. In the zymography assay, MMP9 production was increased following TNF-α treatment and inhibited by LGALS3BP overexpression, whereas MMP2 production was unaffected (Fig. 4E, F). These results show that LGALS3BP overexpression suppresses TNF-α-mediated invasiveness in TNBC by inhibiting MMP9 expression and production.

Together, these findings demonstrate that LGALS3BP interacts with TAK1, thus negatively regulating its phosphorylation and activation during TNF-α treatment, resulting in suppression of MMP9 production and TNBC invasion.

### Synthesis and characterization of LGALS3BP-encapsulated liposomes

**Fig. 5:**
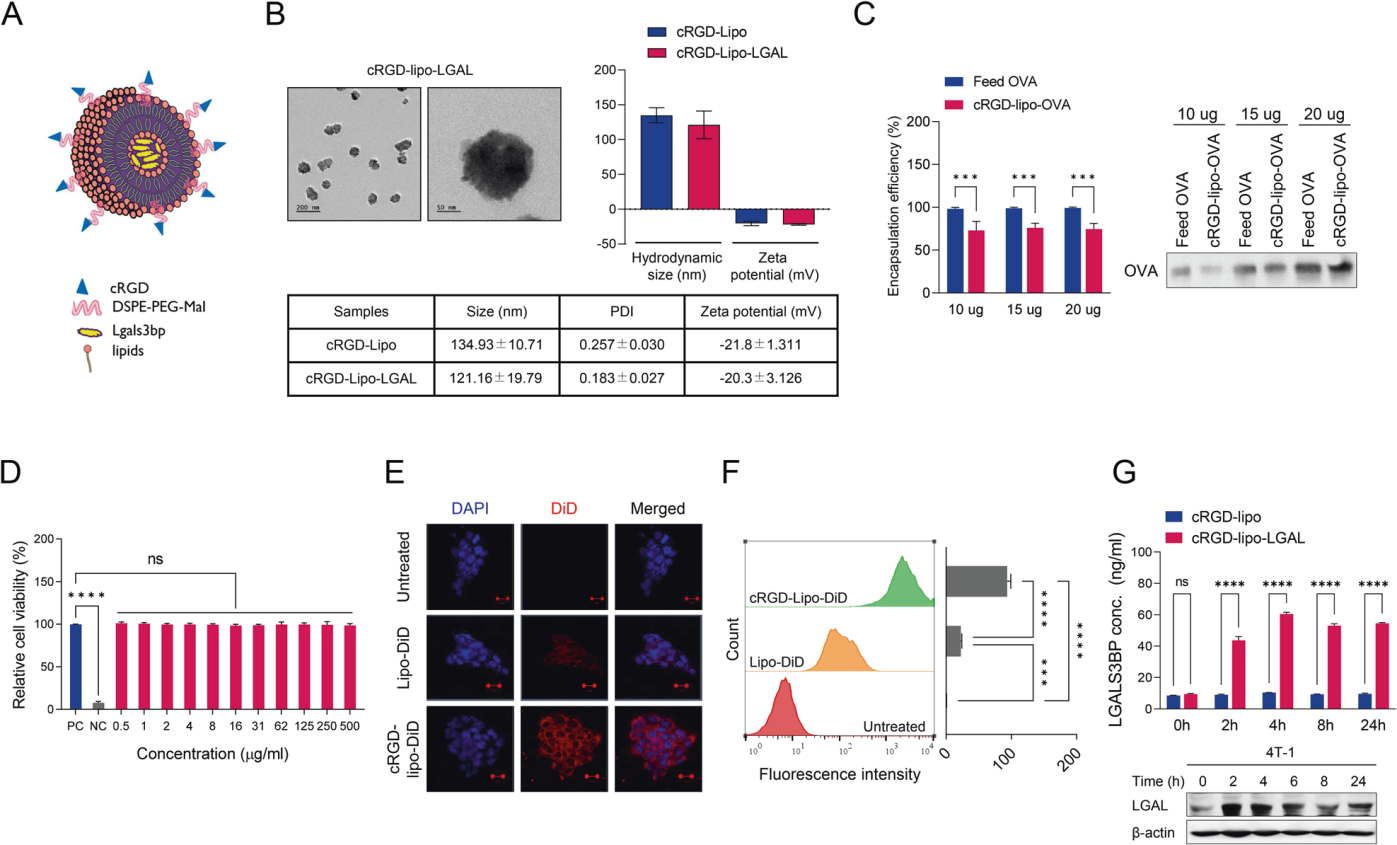
Characterization and cellular uptake of the cRGD-lipo-LGALS3BP nanoparticles. **A** Schematic representation of LGALS3BP-loaded cRGD-liposomes. **B** Characterization of cRGD-liposome-LGALS3BP NPs. Left: TEM images of cRGD-liposome-LGALS3BP (scale bar 200 nm or 50 nm). Lower right: hydrodynamic size and zeta potential of cRGD-liposome and cRGD-liposome-LGALS3BP. **C** Encapsulation efficacy of cRGD-liposome nanoparticles. **D** In vitro cytotoxicity studies of the cRGD-liposome-LGALS3BP. Viability of 4T-1 cells exposed to the cRGD-liposome-LGALS3BP at different concentrations for 24 h of incubation at 37 °C using the WST assay. PC: untreated cells as the positive control, NC: 0.1% Triton X-100-treated cells as the negative control. **E** Fluorescence microscope images of 4T-1 cells incubated by DiD dye (red)-encapsulated liposome, cRGD-liposome, and DAPI (blue). **F** Flow cytometry analysis of liposome-DiD or cRGD-liposome-DiD cellular uptake in 4T-1 cells. Left: representative histograms of the uptake of both liposomes in 4T-1 cells; right: quantitative evaluation of cellular uptake. **G** Evaluation of delivered LGALS3BP levels by liposome in 4T-1 cells treated with cRGD-liposome or cRGD-liposome-LGALS3BP. Upper: ELISA; lower: Western blotting analysis. Data are presented as mean ± SD. *p < 0.05; **p < 0.01; ***p < 0.001; ****p < 0.0001.

Based on The Cancer Genome Atlas dataset, Lgals3bp expression was significantly lower in TNBC than in the other subtypes (Fig. S3). Therefore, we hypothesized that the delivery of LGALS3BP to the TNBC tumor suppresses TAK1 activity, inhibiting TNBC metastasis. We used a cRGD-liposome (hereafter, cRGD-lipo) for encapsulation and in vivo tumor delivery of LGALS3BP. The cRGD-lipo and cRGD-lipo-LGAL nanoparticles had similar hydrodynamic sizes of ~120 and 135 nm, with negative surface charges (zeta potentials) of −22 and −20 mV, respectively (Fig. 5B). Transmission electron microscopy confirmed nanoparticle production. Encapsulation efficiency was approximately 75% (Fig. 5C).

### Cellular uptake of LGALS3BP-encapsulated liposomes

Cytotoxicity was not significant at the concentrations tested (Fig. 5D). Next, we investigated liposome uptake by 4T-1 cells (Fig. 5E). DiD served as a fluorescent agent for the visualization and quantification of cellular uptake of liposomes. The fluorescence of cRGD-lipo-DiD overlapped substantially with that of the cytosol, and cells incubated with cRGD-lipo-DiD nanoparticles exhibited higher fluorescence intensity than those incubated with lipo-DiD nanoparticles. Flow cytometry analysis confirmed that these cRGD-lipo nanoparticles have better cellular uptake than liposomes (Fig. 5F), indicating that cRGD conjugation improved nanoparticle delivery.

We evaluated the nanoparticle delivery of LGALS3BP into 4T-1 target cells. 4T-1 cells treated with cRGD-lipo-LGAL had significantly higher LGALS3BP concentrations than those treated with the cRGD-lipo control (Fig. 5G). These findings indicate that LGALS3BP is effectively delivered into 4T-1 cells by cRGD-liposome and is sustained in the intracellular compartment.

### Nanoparticle biodistribution and tumor targeting

**Fig. 6:**
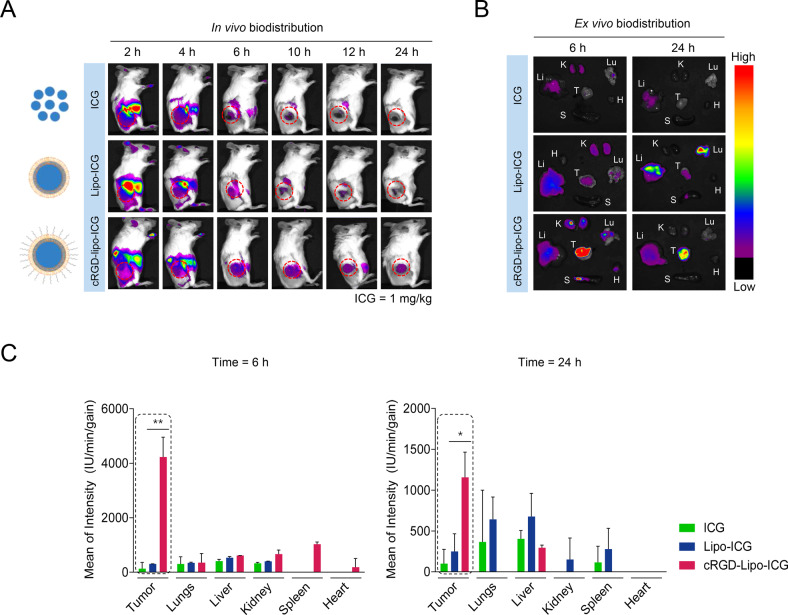
Biodistribution analysis of liposomes. **A** In vivo fluorescence images of ICG, liposome-ICG, and cRGD-liposome-ICG in 4T-1 tumor-bearing mice at indicated times. The red circles indicate the site of the tumors. **B** Ex vivo fluorescence images of tumors and major organs at 6 h and 24 h in the ICG, liposome-ICG, and cRGD-liposome-ICG groups. **C** Quantitative fluorescence results of tumor and major organs (*n* = 3 /group). Data are presented as mean ± SD. *p < 0.05; **p < 0.01; ***p < 0.001; ****p < 0.0001.

To evaluate tumor targeting of nanoparticles, we synthesized and administered cRGD-lipo-ICG into TNBC xenograft mice; ICG served as a near-infrared fluorescent agent for quantifying in vivo localized nanoparticles. cRGD-lipo-ICG exhibited better tumor accumulation than lipo-ICG or ICG dye alone (Fig. 6A). To directly observe nanoparticle accumulation, ex vivo images of major organs and tumors were captured 6 and 24 h post-injection. Consistently, cRGD-lipo-ICG exhibited stronger fluorescence in tumors than in healthy organs (Fig. 6B, C). These findings indicate that the cRGD-liposome nanoparticles are an effective system for delivering LGALS3BP into TNBC tumor tissues.

### Biosafety of cRGD-lipo-LGAL nanoparticles

**Fig. 7:**
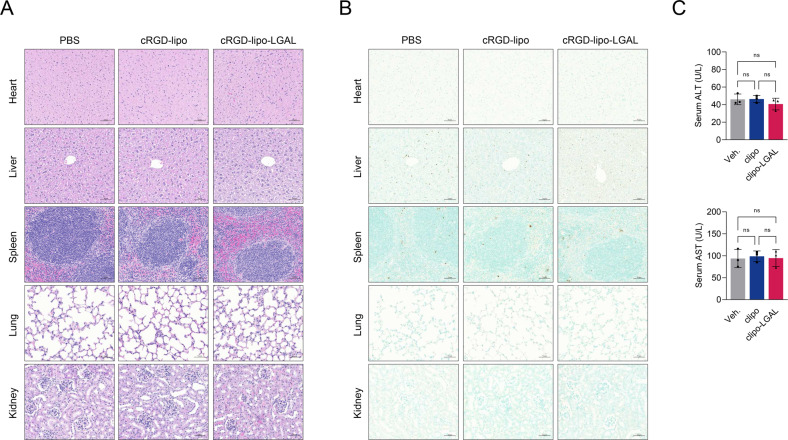
Biosafety evaluation of cRGD-liposome-LGALS3BP. Heart, liver, spleen, lung, and kidney samples were collected from different groups of mice at 24 h post-treatment. **A** Histopathological examination of major organs to assess the biosafety of cRGD-liposome-LGALS3BP. **B** TUNEL staining of major organs following various treatments to evaluate apoptosis. Scale bar: 50 μm. **C** ALT and AST levels of mice in all groups. Data are reported as mean ± SD (*n* = 5 per group). Statistical significance was determined using a two-tailed unpaired *t*-test; ns: statistically insignificant (*p* > 0.05).

Biosafety is important for therapeutic applications of nanoparticles. Therefore, we evaluated cRGD-lipo-LGAL and cRGD-lipo biosafety. Healthy BALB/c mice received PBS as the vehicle, PBS containing cRGD-lipo or cRGD-lipo-LGAL via intravenous administration. Biosafety was evaluated via H&E staining and histological analysis of major organs. Results revealed no tissue damage, and the TUNEL assay further confirmed cRGD-lipo-LGAL biosafety (Fig. 7A, B). Serum ALT and AST levels were not significantly altered (Fig. 7C), suggesting that cRGD-lipo-LGAL is safe.

### Evaluation of the therapeutic effects of cRGD-lipo-LGAL

**Fig. 8:**
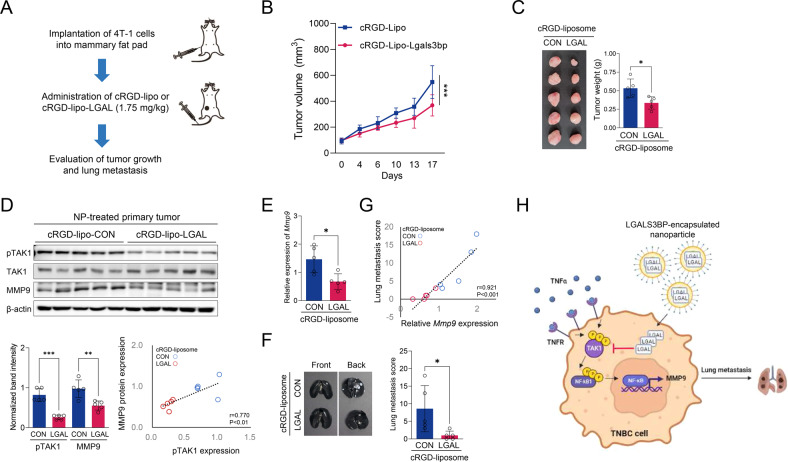
Antitumor efficiency of cRGD-liposome-LGALS3BP in vivo. 4T-1 tumor-bearing mice were administered cRGD-lipo or cRGD-lipo-LGAL (1.75 mg/kg) via intravenous injection for 2 weeks (once a week), as shown in the experimental outline. **A** Experimental outline. **B** The average tumor growth curves after the indicated treatment (*n* = 5 per group). **C** Tumor tissues at the end of antitumor studies. **D** Western blotting analysis of p-TAK1, TAK1, and MMP9 in protein extracts prepared from tumors. Upper: western blotting analysis; lower: quantification of band intensity and the correlation between TAK1 phosphorylation and MMP9 protein expression in whole tumor tissues. **E** Comparison of the mRNA expression of *MMP9* between two groups. **F** Comparison of lung metastasis between two groups. **G** Correlation between gene expression of *MMP9* and lung metastasis score in whole groups. **H** Schematic diagram of the mechanisms of action of cRGD-liposome-LGALS3BP. Data are presented as mean ± SD. *p < 0.05; **p < 0.01; ***p < 0.001.

We investigated whether cRGD-lipo-LGAL nanoparticles inhibit tumor growth and metastasis (Fig. 8A). cRGD-lipo-LGAL administration significantly reduced tumor growth and weight relative to that in cRGD-lipo-treated mice (Fig. 8B, C). Next, we investigated the effect of cRGD-lipo-LGAL administration on TAK1 phosphorylation and MMP9 expression in TNBC primary tumors: both were significantly reduced in the tumor tissues of the cRGD-lipo-LGAL-treated mice, revealing a positive association between TAK1 phosphorylation and MMP9 expression in tumor tissues (Fig. 8D). TNBC lung metastasis, which was positively associated with *MMP9* expression in tumor tissues, was suppressed by cRGD-lipo-LGAL (Fig. 8E–G). cRGD-lipo-LGAL nanoparticles effectively inhibited TAK1 phosphorylation and MMP9 expression, suppressing tumor growth and lung metastasis in TNBC (Fig. 8H).

## Discussion

Chronic inflammation generated by the tumor microenvironment drives cancer progression and metastasis. TNF-α, one of the inflammatory cytokines from the tumor microenvironment, is associated with metastasis and aggressiveness in various cancers, including TNBC [25–28]. TNF-α promotes cancer cell invasion via MMP9 production, which can remodel the extracellular matrix during metastasis [25–27]. In this process, the TAK1 complex is crucial for TNF-α-mediated MMP9 production [29]. Therefore, inhibition of the TNF-α–TAK1–MMP9 axis may be an effective strategy for preventing TNBC metastasis. Here, we revealed that LGALS3BP plays a pivotal role in suppressing the TNF-α–TAK1–MMP9 axis, thereby inhibiting the invasiveness of TNBC.

Accumulating evidence suggests that TAK1 is a key therapeutic target for metastatic BC [30, 31]. As the prevention of aberrant TAK1 activation is important for cancer treatment, there have been numerous attempts to treat cancer using the pharmacological blockade of TAK1 [9–11]. Treatment with 5Z-7-oxozeanenol, a TAK1-specific small molecule inhibitor, suppressed the proliferation of human TNBC cells [11]. Nanoparticles loaded with 5Z-7-oxozeanenol suppressed the lung metastasis of human TNBC in an orthotopic NSG murine model [10]. NG25, another TAK1 inhibitor, enhanced doxorubicin treatment efficacy by increasing doxorubicin-mediated apoptosis in BC cells [32]. In this study, we confirmed that LGALS3BP inhibits TAK1 activation via direct protein–protein interaction in TNBC. Therefore, as an endogenous negative regulator of TAK1, LGALS3BP induction or delivery provides potential therapeutic approaches for TNBC.

In the present study, we attempted to deliver LGALS3BP into TNBC cells and verify its therapeutic effects. Based on improved tumor-targeting ability [22–24], we used cRGD peptide-conjugated liposome as an LGALS3BP-delivery system. Incorporating cRGD facilitated the cellular uptake and tumor-specific targeting of liposomes both in vitro (Fig. 5E, F) and in vivo (Fig. 6). In the orthotopic TNBC mouse model, the cRGD-lipo-LGAL-treated group showed reduced tumor growth and lung metastasis, accompanied by suppressing TAK1 activation and *MMP9* expression, compared to those in the cRGD-lipo control group (Fig. 8). LGALS3BP-derived-peptide-loaded nanoparticles might exhibit greater payload capacity than LGALS3BP protein-loaded nanoparticles, leading to higher therapeutic efficacy in TNBC. Therefore, further studies are required to identify the domain or amino acid sequence of LGALS3BP responsible for TAK1 interaction and inhibition for developing LGALS3BP-derived anti-cancer peptides.

Nevertheless, some questions need to be addressed. First, although we verified the potential of LGALS3BP-loaded nanoparticles as a therapeutic strategy for TNBC, we could not investigate the pharmacokinetics of LGALS3BP-loaded nanoparticles. Further research is required to validate the real-time pharmacokinetics of nanoparticles. Second, our data suggest that LGALS3BP inhibits TAK1 activation via protein–protein interactions. Prior studies have shown that LGALS3BP interacts with TAK1 but not with TAK1 complex adaptor proteins, such as TAB1, TAB2, TAB3, and TRAF6. Furthermore, LGALS3BP-TAK1 interaction reduces the binding affinities between TAK1 and its adaptor proteins. LGALS3BP decreases TAK1 phosphorylation and its kinase activity in a dose-dependent manner [14]. Binding of the adaptor proteins and TAK1 complex formation are required for TAK1 activation [33, 34]. These findings suggest that LGALS3BP reduces the binding affinities between TAK1 and other adaptor proteins through binding competition or masking its binding sites.

In conclusion, nanoparticle-mediated delivery of LGALS3BP could potentially improve TNBC treatment, suppressing cancer progression and metastasis. Our findings revealed the antitumor and anti-metastatic properties of LGALS3BP in TNBC. Mechanistically, LGALS3BP suppressed TNF-α-mediated *MMP9* expression by downregulating TAK1 signaling in TNBC. Nanoparticle-mediated delivery of LGALS3BP exhibited anti-metastatic and antitumor effects, reducing TAK1 phosphorylation and *MMP9* expression in TNBC primary tumor tissues. Therefore, the LGALS3BP-delivery system may provide effective therapy for TNBC and potentially for other cancer types regulated via aberrant TAK1 activation.

## Materials and methods

### Cell culture and transfection

The 4T-1, 4T-1/luc2, and MDA-MB-231 cell lines were obtained from the American Type Culture Collection (Manassas, VA, USA) and Korean Cell Line Bank (Seoul, Korea). Cells were cultured in RPMI-1640 medium or Dulbecco’s modified Eagle’s medium containing 10% fetal bovine serum, 100 units/mL of penicillin, and 100 μg/mL of streptomycin (Invitrogen, Carlsbad, CA, USA) at 37 °C in a humidified atmosphere containing 5% CO_2_. NC siRNA and siMMP9 (sc-29401) were obtained from Santa Cruz Biotechnology (Dallas, TX, USA). DNA and siRNA were transfected using Lipofectamine 3000 (L3000015; Invitrogen) and Lipofectamine RNAiMAX (13778150; Invitrogen), respectively, following the manufacturer’s protocol.

### Plasmid construction

DNA fragments of the mouse *Lgals3bp* and human *LGALS3BP* were PCR-amplified and cloned into pcDNA4/myc-His A or pcDNA6/myc-His A (Invitrogen, Carlsbad, CA, USA) using the In-Fusion HD Cloning kit (Clontech Mountain View, CA, USA), according to the manufacturer’s protocols. The primers used for cloning are listed in Table S1. All constructs were validated using DNA sequencing.

### Reagents

Recombinant TNF-α (210-TA) and LGALS3BP (2226-GAB) proteins and 5Z-7-Oxozeaenol (O9890) were obtained from R&D Systems (Abingdon, UK) and Sigma-Aldrich Co. (St. Louis, MO, USA).

### Animal models and ethical approval

8-10-week old female BALB/c mice were obtained from Orient Bio Inc. (Seongnam, Korea) and maintained in a pathogen-free facility to establish the orthotopic tumor model. Mice were randomly allocated to treatment groups for each in vivo study. All animal care and experiments followed the Institutional Animal Care and Use Committee (IACUC) guidelines. They were approved by the IACUC of Chonnam National University Medical School (Approval No. CNU IACUC-H-2022-6).

### Cell proliferation assay

The cell proliferation assay was performed following the manufacturer’s instructions (G7570; Promega, Madison, WI, USA). Cells were plated onto 96-well white assay plates. After incubation for 24 h, cells were treated with an equal volume of 2X RealTime-Glo reagent, and cell viability was measured using a 96-well plate reader (GloMax-96 microplate luminometer; Promega, Sweden).

### Wound-healing assay

The wound-healing assay was performed using the IncuCyte ZOOM^TM^ system (Essen BioScience, MI, USA) according to the manufacturer’s instructions. Briefly, the cells were seeded and cultured to a 90% confluent monolayer in 96-well ImageLock plates (Essen BioScience). Identical scratches were made using the WoundMaker tool (Essen Biosciences, Hertfordshire, UK). The cells were then washed twice with the medium, and the medium was replenished (100 µL). Plates were incubated in IncuCyte ZOOM^TM^ and photographed every 4 h for 24 h. The scratch closure rate was evaluated using the IncuCyte software, expressed as a percentage of relative wound closure.

### Colony formation assay

4T-1 and MDA-MB-231 cells were seeded on 6-well plates (600 or 300 cells/well) and incubated for 14 d with media change. After washing, cells were fixed with 95% EtOH at 25 °C for 10 min and stained using crystal violet solution (0.1% crystal violet in 1X phosphate-buffered saline (PBS) at 25 °C for 20 min. The number of colonies and area were quantified using ImageJ (National Institutes of Health, Bethesda, MD, USA).

### Invasion assay

Cell invasion ability was measured using the Transwell chamber system. Briefly, cultured cells were seeded onto the top of a 24-well Transwell filter chamber coated with 1 μg/mL Matrigel. A cell culture medium containing 5% bovine serum albumin and 20 μg/mL of fibronectin (Calbiochem, La Jolla, CA) was added to the bottom chamber. After 24 h of incubation, the cells were stained with a Diff-Quick solution (Sysmex, Kobe, Japan). The number or area of invaded cells was calculated using ImageJ (National Institutes of Health).

### RNA-sequencing analysis

One microgram of total RNA was extracted from Lgals3bp-overexpressed 4T-1 cells using a Ribospin RNA extraction kit (GeneAll Biotechnology, Seoul, Korea). Libraries were generated using the TruSeq RNA library prep kit v2 and sequenced on the Illumina platform (all sequencing was carried out by Macrogen, Inc., Seoul, Korea). RSEM with the STAR aligner was used to map mRNA-seq data to the mouse reference genome (version mm10) [35, 36]. Differentially expressed genes (DEGs) were defined as genes with over 2-fold change (FC) in expression between Lgals3bp-overexpressed cells and controls. Analysis of the biological functions of DEGs was conducted via Metascape [37].

### RT-qPCR

Total RNA isolation and cDNA synthesis were conducted using the Hybrid-R reagent (305-101; GeneAll Biotechnology, Korea) and GoScript system (Promega, Madison, WI) following the manufacturer’s protocol. Reverse transcription and quantitative PCR (RT-qPCR) were performed with a CFX96 Real-Time PCR Detection System (Bio-Rad Laboratories, Hercules, CA, USA) using SYBR Green Supermix. The primers used are listed in Table S2. Fold changes were calculated using the ΔΔCT method. The housekeeping gene, *GAPDH*, was used as the internal control.

### Zymography

For zymography, all materials were obtained from Invitrogen. At 70–80% cell confluence, the cell culture medium was replaced with a serum-free medium. After 24 h of incubation, the conditioned medium was collected and loaded onto a sodium dodecyl sulfate-polyacrylamide gel containing gelatin (ZY00100). After electrophoresis, the gels were incubated in 1X zymogram renaturing buffer (LC2670) for 30 min with gentle agitation. After equilibration, the gel was incubated in 1× developing buffer (LC2671) at 37 °C overnight. After staining the gel with the Coomassie G-250 staining buffer (LC6060), band intensity was quantified using ImageJ (National Institutes of Health).

### Immunoprecipitation and western blotting analysis

For immunoprecipitation, cells were lysed with IP lysis buffer (87787; Thermo Fisher Scientific, Waltham, MA, USA). Whole cell lysate (WCL) was incubated with antibodies at 4 °C for 16 h. Protein A/G-agarose beads were added and incubated at 4 °C for 1 h with rotation. After bead washing, immunoprecipitated proteins were analyzed using sodium dodecyl sulfate-polyacrylamide gel electrophoresis and western blotting. For western blotting analysis, WCL was isolated with a protein extraction reagent (78501; Thermo Fisher Scientific). Equal amounts of WCL were resolved using sodium dodecyl sulfate-polyacrylamide gel electrophoresis and transferred onto a polyvinylidene fluoride membrane. Membranes were blocked using SuperBlock^TM^ T20 blocking buffer (37515; Thermo Fisher Scientific) and incubated overnight at 4 °C with primary antibodies (Table S3). Horseradish peroxidase-conjugated secondary antibodies were used to probe the membranes for 1 h at 25 °C, and the membranes were visualized using a low-light imaging system (LAS-4000 mini; Fujifilm, Tokyo, Japan). Band intensities were quantified using the Multi-Gauge 3.0 software (Fujifilm).

### Formulation and characterization of LGAL-loaded cRGD-liposomes

LGALS3BP-loaded cRGD-liposomes were synthesized by the thin-film hydration method. First, 1,2-distearoyl-sn-glycero-3-phosphoethanolamine-N-[maleimide(polyethylene glycol)-2000] (ammonium salt) (DSPE-PEG-Mal, Avanti Polar Lipids, Alabaster, AL, USA) was conjugated to cysteine terminated cRGD peptide (C*GRGDSPK*) through thiol-maleimide conjugation. Dipalmitoyl-sn-glycero-3-phosphocholine (DPPC; Avanti Polar Lipids), cholesterol lipids (Avanti Polar Lipids), and DSPE-PEG-Mal conjugated cRGD at 1.5:1.5:1 weight ratio were combined in a chloroform/methanol mixture and subjected to evaporation to form a thin lipid film. Next, the lipid film was hydrated in 1 mL of LGAL (R&D systems, Abingdon, UK) in PBS for 10 min at 60 °C to form heterogeneous and multivesicular liposomes. The mixture was freeze-thawed for six cycles and then sonicated under the ice for 7 min; the obtained solution was passed through a 200-nm polycarbonate filter fixed in an Avanti mini extruder (Avanti Polar Lipids) for 11 cycles to obtain cRGD-lipo-LGAL. The unloaded components were removed by dialysis (molecular weight cut off = 4–6 kDa). The nanoparticle hydrodynamic size (DLS analysis) and zeta potential were assessed using a Zetasizer Nano Z instrument (Malvern Instruments, Malvern, UK). The morphology of the nanoparticles was analyzed via field-emission transmission electron microscopy (FE-TEM) (JEM-2100F JEOL, Tokyo, Japan). A model protein ovalbumin was used instead of LGALS3BP to calculate the encapsulation efficiency of nanoparticles. The encapsulation efficiency was calculated based on the quantification data obtained from western blotting analysis of cRGD-lipo-ovalbumin for varying feed concentrations of ovalbumin.

### Cytotoxicity and cellular uptake assay

Cell viability was measured via the WST assay. To investigate the cellular uptake of cRGD-lipo-LGAL, DiD was used instead of LGAL for visualization and quantification. Briefly, cRGD-lipo-DiD (DiD concentration: 2 μg mL^−1^) and lipo-DiD were added into 4T-1 cells in Lab-Tek® Chamber Slide. After 6 h of incubation, cells were fixed and stained with 4′,6-diamidino-2-phenylindole. Cells were subjected to confocal microscopy and flow cytometry analysis. The cellular uptake of cRGD-lipo-LGAL was analyzed via an enzyme-linked immunosorbent assay (ELISA, IBL, Gunma, Japan, Cat#: 27796).

### In vivo biodistribution

Fluorescent dye indocyanine green (ICG), lipo-ICG, and cRGD-lipo-ICG (1 mg kg^−1^ ICG) were injected intravenously into 4T-1 tumor-bearing mice. Biodistribution was analyzed using a fluorescence-labeled organism bio-imaging instrument (FOBI, Neo-Science, Gyeonggi, Korea).

### Pulmonary metastatic lesion evaluation

The India ink assay was used to quantify lung metastasis. Pulmonary metastases were visualized following intratracheal injection of 5 mL India ink (15% v/v diluted in water; Hardy Diagnostics, Santa Maria, CA, USA). The lungs were then washed in Fekete’s solution (300 mL 70% EtOH, 30 mL 37% formaldehyde, 5 mL glacial acetic acid) and placed in fresh Fekete’s solution overnight at 4 °C. The pulmonary metastatic nodules were observed as white spots on the black lung tissue background and counted manually.

### Measurement of serum ALT and AST levels

Alanine aminotransferase (ALT) and aspartate aminotransferase (AST) levels were determined through blood biochemistry analysis using an automated blood biochemistry analyzer (Catalyst One, IDEXX Laboratories, Inc., Westbrook, ME, USA), following the manufacturer’s instructions.

### Histological studies

Major organ sections were stained with hematoxylin, eosin, or terminal deoxynucleotidyl transferase dUTP nick end labeling (TUNEL) from T&P Bio (Gyeonggi, Korea). The slides were imaged using a Zeiss Axio Scan.Z1 slide scanner (Carl Zeiss, Jena, Germany).

### Statistical analysis

All experiments were performed in triplicate unless otherwise noted. Data are presented as mean ± standard deviation (SD). Statistical significance was assessed using the Student’s *t*-test for comparison between two groups and one-way analysis of variance (ANOVA) for multiple groups (GraphPad Software, La Jolla, CA, USA). Statistical significance was set at *P* < 0.05.

## Supplementary information

Supplemental information

Supplementary figure 1

Supplementary figure 2

Supplementary figure 3

Supplementary figure 4

## Data Availability

The data sets that support the findings of the current study are available from the corresponding authors upon reasonable request.
