# Supplementary material for: Suppression of triple-negative breast cancer aggressiveness by LGALS3BP via inhibition of the TNF-α–TAK1–MMP9 axis
Source: Cell Death Discov. 2023 Apr 11;9:122. doi: 10.1038/s41420-023-01419-9 (PMC10090165; doi:10.1038/s41420-023-01419-9)
Supplement: Supplementary file 1 — Supplemental information [file 41420_2023_1419_MOESM1_ESM.docx]

**Supplementary Information**

**Supplementary Tables**

**Table S1: Primers used for plasmid construction.**

| Primer name | Sequence (5′-3′) | Vector |
| --- | --- | --- |
| mLGAL3BP-F | ACCGAGCTCGGATCCATGGCTCTCCTGTGGCTCCTCTCT | pcDNA4/myc-His A |
| mLGAL3BP-R | GAAGGGCCCTCTAGACACCATGTCAGTGGAGTTAGT |  |
| hLGAL3BP-F | AGGTACCTAGGATCCATGACCCCTCCGAGGCTCTTC | pcDNA6/myc-His A |
| hLGAL3BP-R | AACGGGCCCTCTAGAGTCCACACCTGAGGAGTTGG |  |

Underlined sequences represent complementary sequences and contain restriction enzyme sites.

**Table S2: Primers used for RT-qPCR.**

| Gene | Primer sequence | |
| --- | --- | --- |
|  | Forward (5′-3′) | Reverse (5′-3′) |
| mMMP9  (NM_013599) | GCGGACATTGTCATCCAGTTTG | CGTCGTCGAAATGGGCATC |
| hMMP9  (NM_031055) | GCCACTACTGTGCCTTTGAGTC | CCCTCAGAGAATCGCCAGTACT |
| mTNF-α  (NM_013693) | AGGGTCTGGGCCATAGAACT | CCACCACGCTCTTCTGTCTAC |
| mIL-6  (NM_031168) | CCACCACGCTCTTCTGTCTAC | ACCAGAGGAAATTTTCAATAGGC |
| mGAPDH  (NM_008084) | AGGTCGGTGTGAACGGATTTG | TGTAGACCATGTAGTTGAGGTCA |
| hGAPDH  (NM_002046) | GGCTCTCCAGAACATCATC | TCCACCACTGACACGTTG |

**Table S3: Primary antibodies used in the study.**

| Antibody | Manufacturer | Cat. No. | Application | Dilution |
| --- | --- | --- | --- | --- |
| TAK1 | CST | 5206 | WB | 1:1000 |
| TAK1 | Abcam | ab109526 | IP  WB | 2 µg/test  1:1000 |
| p-TAK1 | Abcam | ab109404 | WB | 1:1000 |
| p-TAK1 | Millipore | 06-1425 | WB | 1:1000 |
| p-IKKαβ | CST | 2697 | WB | 1:1000 |
| p-NF-kB p65 | CST | 3033 | WB | 1:1000 |
| NF-kB p65 | CST | 6956 | WB | 1:1000 |
| p-IkBα | CST | 9246 | WB | 1:1000 |
| IkBα | CST | 4814 | WB | 1:1000 |
| Myc | MBL | M192-3 | IP, WB | 2 µg/test |
| Myc | MBL | 562 | WB | 2 µg/test |
| Lgals3bp | IBL | 28125 | WB | 1:1000 |
| MMP9 | Abcam | ab228402 | WB | 1:1000 |
| MMP9 | Novous | NBP57940 | WB | 1:1000 |
| β-actin | Abcam | ab6276 | WB | 1:1000 |
| β-actin | Abcam | ab8227 | WB | 1:1000 |
| Anti-mouse Ig HRP | Rockland | 18-8817-33 | WB | 1:1000 |
| Anti-Rabbit Ig HRP | Rockland | 18-8816-33 | WB | 1:1000 |

All secondary HRP-conjugated antibodies for western blotting analysis were purchased from Jackson ImmunoResearch Laboratories. Secondary HRP-conjugated antibodies produced by Rockland were used for western blotting analysis of immunoprecipitated proteins. Abbreviations: WB, Western blot; IP, Immunoprecipitation; CST, Cell Signaling Technology.

**Supplementary Figures**

**Fig. S1: Confirmation of stable cells. Extended data related to Figure 1.** After establishing stable cells, the efficiency of overexpression was determined at both the mRNA and protein levels using RT-qPCR and western blotting analysis, respectively. Comparison of mRNA (A, C) and protein levels (B, D) of 4T-1 cells and MDA-MB-231 cells.

**Fig. S2: LGALS3BP suppresses *in vivo* tumor growth and lung metastasis. Extended data related to Figure 2.** Non-labeled parent 4T-1/EV or 4T-1/LGAL cells were inoculated into the mammary fat pad of BALB/c mice (n = 5/group). (A) Average tumor growth curves. (B) Comparison of tumor weight between the 4T-1/EV and 4T-1/LGAL-inoculated groups. (C) Comparison of spleen weight. (D) Comparison of lung metastasis in BALB/c mice injected with 4T-1/EV and 4T-1/LGAL.

**Fig. S3: TCGA database (available from cBioportal) for *LGALS3BP* expression in human breast cancers.** A comparison of mRNA expression of *LGALS3BP* between non-TNBC and TNBC was obtained from The Cancer Genome Atlas (TCGA) dataset based on the breast cancer project: 1605 non-TNBC tissues and 299 TNBC tissues.

**Fig. S4: Input control for LGALS3BP and TAK1 immunoprecipitation. Extended data related to Figure 4.** (A) Input control. (B) Uncropped western blot images.
