# Supplementary figures and images for: Suppression of triple-negative breast cancer aggressiveness by LGALS3BP via inhibition of the TNF-α–TAK1–MMP9 axis
Source: Cell Death Discov. 2023 Apr 11;9:122. doi: 10.1038/s41420-023-01419-9 (PMC10090165; doi:10.1038/s41420-023-01419-9)

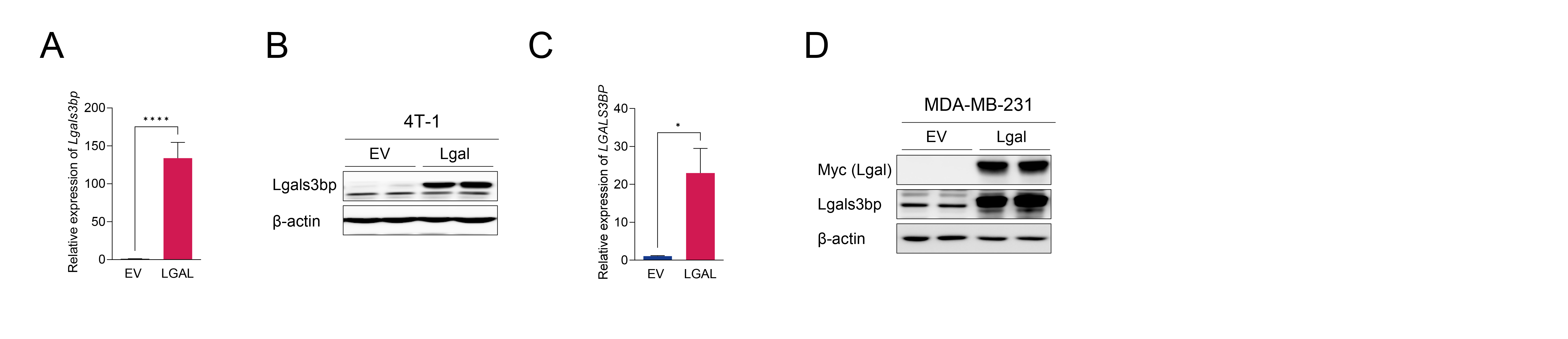

Supplement: Supplementary file 2 — Supplementary figure 1 [file 41420_2023_1419_MOESM2_ESM.tif]

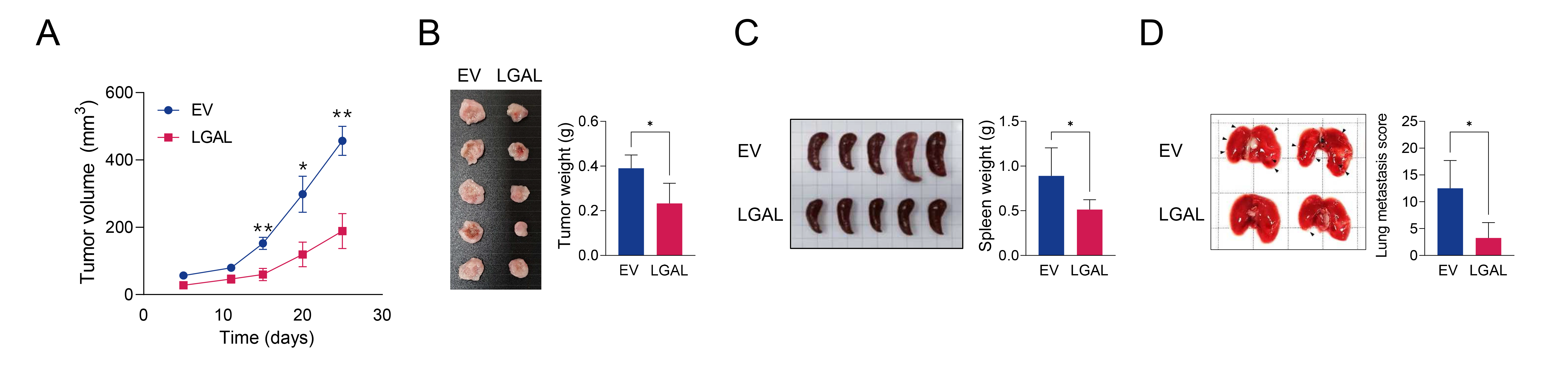

Supplement: Supplementary file 3 — Supplementary figure 2 [file 41420_2023_1419_MOESM3_ESM.tif]

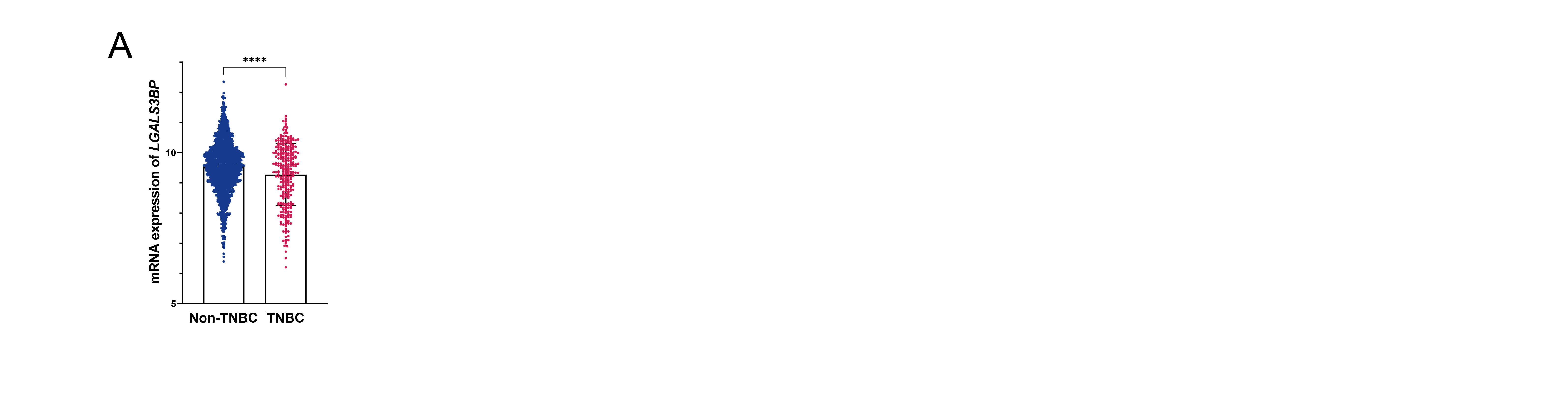

Supplement: Supplementary file 4 — Supplementary figure 3 [file 41420_2023_1419_MOESM4_ESM.tif]

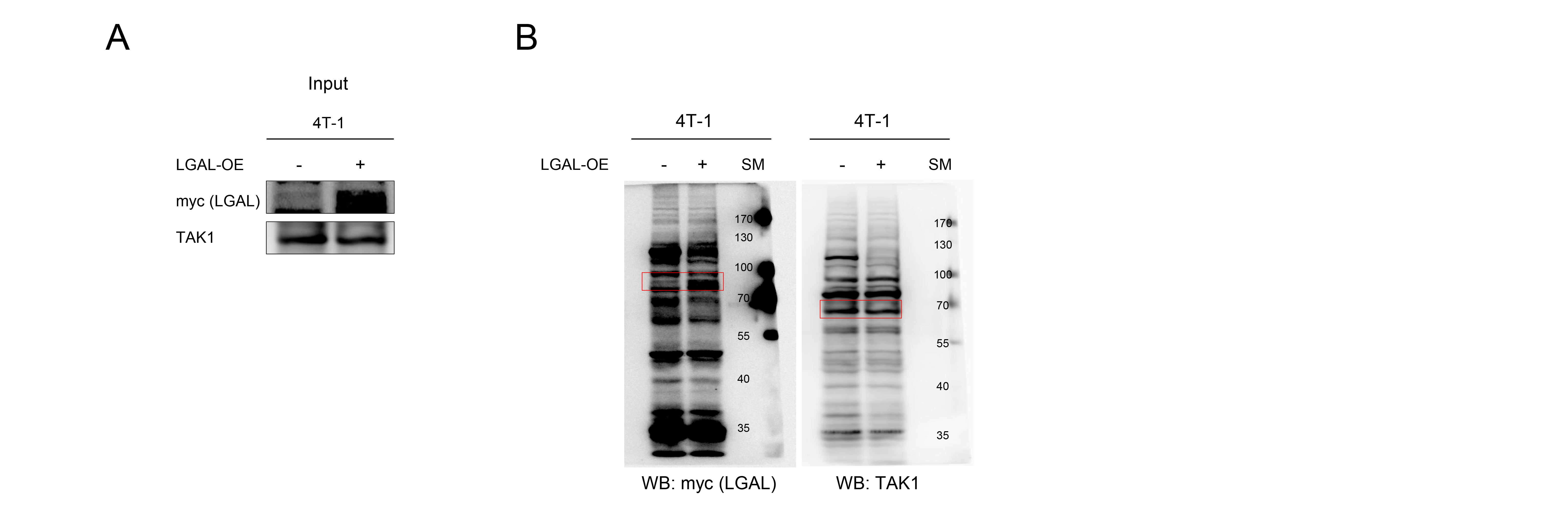

Supplement: Supplementary file 5 — Supplementary figure 4 [file 41420_2023_1419_MOESM5_ESM.tif]
